# Deforestation‐free land‐use change and organic matter‐centered management improve the C footprint of oil palm expansion

**DOI:** 10.1111/gcb.16069

**Published:** 2022-01-21

**Authors:** Juan Carlos Quezada, Thomas Guillaume, Christopher Poeplau, Jaboury Ghazoul, Alexandre Buttler

**Affiliations:** ^1^ Agroscope Field‐Crop Systems and Plant Nutrition Nyon Switzerland; ^2^ Laboratory of Biogeosciences Institute of Earth Surface Dynamics University of Lausanne Lausanne Switzerland; ^3^ Thuenen Institute of Agricultural Climate Research Braunschweig Germany; ^4^ Chair of Ecosystem Management Institute of Terrestrial Ecosystems Department of Environmental Systems Science ETHZ Zürich Switzerland; ^5^ Prince Bernhard Chair for International Nature Conservation, Ecology and Biodiversity Utrecht University Utrecht The Netherlands; ^6^ Centre for Sustainable Forests and Landscapes University of Edinburgh Edinburgh Scotland; ^7^ Laboratory of Ecological Systems ECOS and Plant Ecology Research Laboratory PERL School of Architecture Civil and Environmental Engineering ENAC École Polytechnique Fédérale de Lausanne EPFL Lausanne Switzerland; ^8^ Swiss Federal Institute for Forest, Snow and Landscape Research WSL Lausanne Switzerland

**Keywords:** biomass, C sequestration, degraded savannas, ecosystem C stocks, savanna, soil fertility, soil organic carbon, δ^13^C

## Abstract

In recent decades, mounting evidence has indicated that the expansion of oil palm (OP) plantations at the expense of tropical forest has had a far pernicious effect on ecosystem aspects. While various deforestation‐free strategies have been proposed to enhance OP sustainability, field‐based evidence still need to be consolidated, in particular with respect to savanna regions where OP expansion has recently occurred and that present large area with potential for OP cultivation. Here we show that the common management practice creating within the plantation the so‐called management zones explained nearly five times more variability of soil biogeochemical properties than the savanna land‐use change per se. We also found that clayey‐soil savanna conversion into OP increased total ecosystem C stocks by 40 ± 13 Mg C ha^−1^ during a full OP cultivation cycle, which was due to the higher OP‐derived C accumulated in the biomass and in the soil as compared to the loss of savanna‐derived C. In addition, application of organic residues in specific management zones enhanced the accumulation of soil organic carbon by up to 1.9 Mg ha^−1^ year^−1^ over the full cycle. Within plantation, zones subjected to organic amendments sustained similar soil microbial activity as in neighboring savannas. Our findings represent an empirical proof‐of‐concept that the conversion of non‐forested land in parallel with organic matter‐oriented management strategies can enhance OP agroecosystems C sink capacity while promoting microbe‐mediated soil functioning. Nonetheless, savannas are unique and threatened ecosystems that support a vast biodiversity. Therefore, we suggest to give priority attention to conservation of natural savannas and direct more research toward the impacts of the conversion and subsequent management of degraded savannas.

## Graphical abstract

Alternative land‐use changes like degraded savannas conversion holds potential to replace tropical deforestation and its pernicious effect on ecosystem aspects. However, lack of empirical evidence hinders the understanding of the impacts of such alternative pathway for sustainable agricultural development on important ecosystem processes. Here, we show that tropical savanna conversion into oil palm plantations are a viable option to reduce or even avoid C emissions and other soil degradation processes. While the potential is high for converting degraded savannas, we also warn that preserving natural savanna areas is paramount to ensure the sustainability of oil palm agriculture.

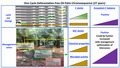

## Opsomming

En décadas recientes, creciente evidencia indica que la expansión de las plantaciones de palma aceitera a expensas de los bosques tropicales ha tenido efectos perniciosos sobre varios aspectos del ecosistema. Si bien se han propuesto varias estrategias libres de deforestación para mejorar la sostenibilidad de las plantaciones de palma aceitera, aún se necesita consolidar evidencia de campo, en particular con respecto a regiones de sabana donde se ha producido recientemente la expansión de la palma aceitera y que presentan una gran área con potencial para el cultivo de la misma. Aquí demostramos que la prácticas de manejo común en plantaciones de palma que crean dentro de las mismas las conocidas zonas de manejo explicó casi cinco veces más la variabilidad de las propiedades biogeoquímicas del suelo en comparación al cambio de uso de la tierra de la sabana per se. También encontramos que la conversión de sabanas de suelo arcilloso en plantaciones de palma aceitera aumentó los stocks totales de C del ecosistema en 40 ± 13 Mg C ha‐1 durante un ciclo completo de cultivo de palma aceitera. Esto se debió al mayor C acumulado en la biomasa de la palma y el C derivado de la palma en el suelo, en comparación con la pérdida de C derivado de la sabana en el suelo. Además, la aplicación de residuos orgánicos en zonas de manejo específicas mejoró la acumulación de C orgánico del suelo en hasta 1.9 Mg ha‐1 año‐1 durante el ciclo completo. Dentro de la plantación, las zonas sometidas a enmiendas orgánicas mantuvieron una actividad microbiana del suelo similar a la de las sabanas vecinas. Nuestros hallazgos representan una prueba de concepto empírica de que la conversión de tierras no boscosas en paralelo con estrategias de manejo orientadas a la materia orgánica pueden mejorar la capacidad de sumidero de C de los agroecosistemas palmeros, al tiempo que promueve el funcionamiento microbiano del suelo. Sin embargo, las sabanas son ecosistemas únicos y amenazados que sustentan una vasta biodiversidad. Por lo tanto, sugerimos dar atención prioritaria a la conservación de las sabanas naturales y dirigir más investigaciones hacia los impactos de la conversión y posterior manejo de sabanas degradadas.

## 1 INTRODUCTION

Understanding the impacts of land‐use change and subsequent agricultural management in the tropics is of increasing pertinence given their importance as drivers of environmental and climatic change (Harris et al., 2012; Röll et al., 2019; Wilcove et al., 2013). Agricultural expansion on formerly forested land has been a particular issue of concern. Tropical deforestation is associated with around 60% loss in ecosystem C storage when forests are replaced by perennial plantations such as oil palm (OP). Such carbon losses contribute to climate change and pose challenges for the sustainability of agroecosystems (Guillaume et al., 2018; Ziegler et al., 2012), particularly as declining soil C reduces soil fertility and biological activity (Don et al., 2011; van Straaten et al., 2015). This cast doubts on the soils’ capacity to sustain OP cultivation on the long term (Guillaume et al., 2016; Kooch et al., 2018; Kopittke et al., 2017; Maranguit et al., 2017). From an ecosystem C standpoint, alternatives to the expansion of OP onto forested land should include the establishment of OP and other perennial crops on low‐biomass land with low soil organic C (SOC; Gibbs et al., 2008; Ziegler et al., 2012). Such alternatives include the conversion of former croplands (i.e., bananas; Furumo & Aide, 2017) or, when appropriate, other non‐forested ecosystems including degraded savannas (Goodrick et al., 2015; Quezada et al., 2019). In South America, initiatives to decouple the expansion of agriculture from deforestation include the 2017 zero‐deforestation agreement for palm oil production established between the Colombian government and the private OP sector (TFA 2020, 2017).

Expansion of OP in Colombia occurs with little direct impact on forests (Furumo & Aide, 2017; Vijay et al., 2016), which is in contrast to past trajectories of OP development in Southeast Asia. A large share (ca. 45%) of the total expansion of OP in Colombia has occurred in the savanna region of Los Llanos in eastern Colombia, and future expansion in this area is predicted to continue (Castiblanco et al., 2013; Etter et al., 2010). Colombian savannas have acidic soils with poor nutrient and soil organic matter content, and high aluminum toxicity (Basamba et al., 2006; Guimarães et al., 2004). Paradoxically, such inherently poor savanna soils are often cited as the last frontier for agricultural expansion given their vast spatial extents in the tropics and their flat topography, which make them suited for large scale agriculture (Ayarza et al., 2007; Guimarães et al., 2004; Rudel et al., 2015). Natural savannas are recognized to have inherent value as providers of a range of hydrological services and as habitats for many native plant and animal species. Yet, many of these savannas have long been exposed to extensive cattle pastures (at around 0.2 head ha^−1^) with a more recent trend of intensification toward improved pastures sown with maize (often with fertilizer application) that support one or more head of cattle per hectare (Vera & Hoyos Garcés, 2019). Even these more intensive cattle pasture systems have marginal viability in the Llanos region and there has been a shift toward other land uses including OP cultivation (Garcia‐Ulloa et al., 2012; Romero‐Ruiz et al., 2012). In the current midst of global climate crisis, using low biomass ecosystems, such as degraded savanna systems, for OP agriculture instead of forests offers opportunities for climate change mitigation by sequestering C in aboveground biomass, and also through soil C sequestration and storage (Laganière et al., 2010; Sanderman et al., 2017).

Following conversion of grazed savanna to new agricultural land uses (often including crops such as maize or rice, as well as OP), subsequent management is an important driving factor determining the direction, magnitude, and rate of change of a wide range of soil properties and processes including nutrient cycling, carbon storage and biological activity. For example, adding crop residues can increase SOC and soil fertility, and improve degraded savanna soils (Rhebergen et al., 2020; Tao et al., 2016, 2017). Fine‐scale spatial differences in soil properties are common in OP plantations due to the distribution of litter and other crop residue inputs as well as fertilizer within plantations (Nelson et al., 2014; Rüegg et al., 2019). This non‐random distribution of nutrient resources within plantations should be considered when assessments of soil properties and processes are attempted (Goodrick et al., 2016; Nelson et al., 2015). With time, the addition of carbon and nutrient crop residue inputs as well as fertilizers in certain areas of the plantation creates management zones that become visually recognizable in mature plantations of around 6–8 years. This fine‐scale within‐plantation heterogeneity among management zones offers an ideal study system for assessing the responses of soil properties and processes to different but consistent management interventions and to outline management practices that have the potential to increase stabilization of OP‐derived C and more generally soil sustainability.

Soil properties and processes differ across OP plantation management zones. Although the impacts of management in OP plantations on soil physical and chemical aspects have been widely studied (Frazão et al., 2013; Khasanah et al., 2015; Quezada et al., 2019), information on its effects on the processes influencing SOC stabilization is limited. In particular, it remains inconclusive how the long‐term management interventions at the OP management zones affect SOC stabilization, that is, effects of cumulative organic matter or nutrient inputs in old plantations. The temporal patterns of soil microbial activity and how this affects SOC stabilization remains also largely unknown. At present, estimates of SOC cycling from savanna conversion to OP are limited because almost all research work has been focused on the more common conversion pathway from forest to OP. In this context, recent work has shown that conversion of pastures in savanna areas, to OP can be C neutral at the ecosystem level (Quezada et al., 2019). It is therefore relevant to assess the environmental impacts of direct savanna conversion into OP.

The overarching aim of this study was to provide a proof‐of‐concept that deforestation‐free alternative of OP cultivation can result in a positive ecosystem C budget. We therefore assessed the effect of savanna conversion to OP on ecosystem C storage and explored how the common OP management practice, reflected in the different management zones, could improve the C footprint of this conversion pathway and maintain soil functioning. We quantified changes in ecosystem C stocks, including the C pools in the vegetation and in the soil, down to 70 cm, using a chronosequence of OP plantations that expand over a time frame of a full OP cultivation cycle (27 years). We hypothesized that OP cultivation on former savanna areas would lead to a positive ecosystem C balance due to the higher aboveground biomass in OP than in savanna vegetation, combined with a limited loss of soil carbon. Furthermore, using the gradual change in the natural abundance of ^13^C due to the land‐use change from savanna (C_4_ vegetation type) to OP (C_3_ vegetation type), we aimed at assessing SOC dynamics in the management zones as we expected different rates of new OP‐derived C accumulation and of savanna‐derived C decomposition, with highest SOC storage in zones of crop residue accumulation. Finally, we also aimed at characterizing changes in soil biogeochemical properties for each management zone as mineral fertilization and plant residue application may strongly affect soil microbial activity and, in turn, soil C cycling. For this, our third hypothesis was that along with increases in soil fertility, soil microbial functioning measured by enzymatic activity and calculated indices will be enhanced.

## 2 MATERIALS AND METHODS

### 2.1 Study area and design

The study area was located at ~20 km east of Puerto Gaitán in the Department of Meta, Colombia, in the slightly undulating well‐drained high plains savannas region of Los Llanos (Altillanura plana). The region experiences a tropical climate (mean annual temperature of 26°C and annual precipitation of 2200 mm year^−1^), with a distinct dry season from December to March and 95% of the yearly rain falling between April and November (Lavelle et al., 2014; Rippstein et al., 2001). The dry season is well marked and affects OP oil yield, which is about 20% less in this savanna region as compared to other regions of Colombia. Soils are dominated by Plinthosols and Ferralsols (IUSS Working Group WRB, 2014). Low fertility, high acidity and high aluminum saturation limiting agricultural production are predominant characteristics of these savanna soils (Lavelle et al., 2014; Rippstein et al., 2001). These savannas are regularly burned, that is, once every or every other year, to provide better forage for the cattle (Romero‐Ruiz et al., 2010). This perturbation operates a selection of plant species resistant to fire, mainly grasses and small shrubs, and prevent a return to tropical rainforest. Their vegetation is characterized by C4 tropical grasses (mainly *Andropogon* and *Trachypogon* grass species) and small scattered patches of fire‐resistant shrub species. The research sites were chosen following a large field survey of about 20 large‐scale OP operations across the savanna region of Los Llanos in Colombia. Those visited farms represent about 20% of all OP plantations in the region (30,000 ha). Following this large survey, and based on records provided by farm agronomists, we deliberately excluded all those sites with steep slopes, inundated parts or located on floodplains, and with distinct past land use to savanna or different management to the standard practices in the region.

All selected OP sites were within the large‐scale commercial farm of “Sapuga” (4°4′N, 72°0′W, 175 km due east of the town of Villavicencio) of about 3000 ha in the Los Llanos savanna area of Colombia. The two reference savanna sites were in the vicinity of Sapuga farm. The OP plots were selected within the OP sites, which had between 20 and 30 ha. They were selected with the aim of evaluating changes in soil characteristics following savanna conversion and therefore they were carefully chosen with the help of plantation agronomists to ensure that the investigated plantations had been established on savannas. A total of four OP plantation sites were identified, with time after savanna conversion at these sites being 8, 12, 23 and 27 years. Also, to minimize biased estimates by variations in soil clay content, all the study sites were established on clayey soil of between 38% and 48% clay content (topsoil 0–10 cm). We recognize that the Colombian Llanos savanna can be variable in biophysical traits at regional scales (Etter et al., 2010), but our site selection sought to minimize such variation to allow for the space‐for‐time approach to test the idea that alternative land‐use change pathways might have practical application from a carbon mitigation potential. The fertilization regime included two applications per year during the early and middle stages of rainfall periods. At plantation establishment, 2–3 tons ha^−1^ of a mix of lime dolomite with phosphoric rock are applied. Depending on plantation age between 150 and 600 kg ha^−1^ year^−1^of NPK together with other macronutrients or micronutrients such as magnesium, boron and zinc are applied. For example, in mature plantations (8–23 years), usual application rates were of 128 kg of N ha^−1^ year^−1^, 18 kg of P ha^−1^ year^−1^ and 249 kg of K ha^−1^ year^−1^.

### 2.2 Soil sampling and sample preparation

At each OP site of 20–30 ha, a representative (i.e., soils, management, fertilization) 1‐ha plot with OP density of 144 palms ha^−1^ was selected. Within each plot, 10 palm trees were randomly selected for referencing soil sample collection and to account for spatial variability. The management zones in OP plantations include (1) the weeded circle (W), a circular bare surface close to the OP trunk of around 4 m diameter, where fertilizers are exclusively applied in early plantation development (1–5 years old); (2) the frond pile (F), where almost all aboveground C inputs are derived from stacked pruned fronds, and where some fertilizer is also added when plantations are between 6 and 30 years; (3) bare soil harvest paths (H) alongside every second row of OP, where farm machinery circulates and from where fertilizers are evenly spread in mature and old plantations (6–30 years); and (4) the interzone (IZ), the area between rows of planted OPs that alternates with the H zone and where understory vegetation grows sporadically (Figure S1). Soils were sampled in the four management zones around each selected palm (see Figure S1) and mixed instead of keeping them separated to avoid pseudo‐replication. Samples were collected with a 6 cm diameter corer to a depth of 70 cm, with intervals of 0–5, 5–10, 10–20, 20–30, 30–50 and 50–70 cm. Litter layer was absent in savannas and OP plantations except at F where superficial fragmented litter was carefully removed by hand until the organo‐mineral soil was exposed.

Savanna sites were sampled along a 100 m long transect with five evenly spaced sampling points. Savanna grasses were flowering at sampling time and were therefore approximately at their maximum biomass stage. As management zones do not occur in savannas, final samples were pooled by depth, as for the OP samples, for each of the two sampled sites for savannas. After field collection and before further analyses, soil samples were homogenized, air‐dried, sieved through a 2 mm stainless steel sieve and stored in plastic bags at room temperature until transportation to the laboratory in Lausanne, Switzerland. This ensured a homogenization of the conditions for all samples that enables the comparison among them without bias due to contrasting soil wetness conditions at the different sampling times or introducing variability due to various storage time and conditions. For soil biological analysis, dried soil samples were subsequently rewetted to 60% of their water‐holding capacity, which provides a measure of the potential of the soil to sustain microbial activity rather than the active pool at the time of field sampling. For soil chemical analysis, samples were oven‐dried at 35°C and analyzed within ~100 days after field collection.

Soil bulk density samples were collected down to 70 cm depth in one soil pit at each 1‐ha OP plot. The pit was dug in the IZ area given the large proportion that it represents at the plot level (Table 1). For the savanna sites, bulk density samples were taken from a pit dug in a mid‐position of the 100 m long transect traced for soil sample collection. At all sites, two stainless steel cylinders volume cores (100 cm^3^) were horizontally inserted in the middle of each sampling layer. Bulk densities were also determined for the topsoil layers (0–5 cm) in the other three management zones. Collected samples were oven‐dried at 105°C for 48 h. Average core weights were recorded and used to obtain mean bulk density values per soil layer. For the SOC stocks calculation in the layers below 5 cm in W, F and H management zones, bulk density values obtained from the pit's deeper layers in IZ were used.

**TABLE 1:** Bulk and OP‐derived SOC accumulation rates at the weeded circle (W), frond piles (F), interzone (IZ) and harvest path (H) management zones in the entire soil profile (0–70 cm) over a full rotation cycle of 27 years

Management zone	Proportion of the total plantation area (%)	Accumulation rates (Mg ha^−1^ year^−1^)	OP‐derived accumulation rates (Mg ha^−1^ year^−1^)
W	12	1.9 (0.3)	2.4 (0.3)
F	11	1.8 (0.6)	2.1 (0.3)
IZ	38	NS	0.8 (0.2)
H	39	NS	0.6 (0.2)

Abbreviation: NS, not significant; OP, oil palm; SOC, soil organic carbon.

### 2.3 Aboveground biomass carbon stocks

Aboveground biomass estimates were conducted by measuring the heights of the 10 randomly selected palms within the 1‐ha plot from the palm base to the base of the youngest fully expanded leaf (Kotowska et al., 2015). Aboveground biomass estimation was done with the use of the allometric equation (Khasanah et al., 2015):

AGBOP=0.0923∗height[m]+0.1333.

Belowground biomass in OP was estimated though allometric equation (Göttingen & Syahrinudin, 2005):

$$\text{BGB}\;\text{OP}=1.45*\text{age}+9.88*144\;\text{palms}\;{\text{ha}}^{‐1}.$$

A factor of 0.413 was used to convert aboveground and belowground biomass to estimate biomass C stocks (Göttingen & Syahrinudin, 2005).

For savanna aboveground biomass measurement, herbaceous vegetation was sampled from one square meter in triplicates in each savanna, close to the transect where soils were sampled. Savanna belowground biomass was sampled at five sampling points in each savanna with a corer of 5 cm diameter to a depth of 30 cm. Roots were separated from the soil by sieving at 2 mm and rinsing. Dry belowground biomass was determined after 48 h of drying at 60°C. Other ecosystem C components such as litter layer in savannas were negligible (Batlle‐Bayer et al., 2010). All biomass measures are given in Mg C ha^−1^. Total biomass time‐averaged C (aboveground biomass+belowground biomass) in OP plantations was estimated as the stocks accumulated in half the time of a rotation cycle in a 30‐year plantation. In OP‐producing countries, plantations are generally kept for several cycles, which justifies the time‐averaged calculation approach. Once a plantation is terminated, new OP trees are planted in the same cultivated area and a new 25‐ to 30‐year cycles starts. Time‐averaged total ecosystem C stocks resulted from summing the total time‐averaged biomass and mean soil C stocks to a depth of 70 cm across all sites. Mean soil C stocks across sites were used because no change over cultivation time were detected. This time‐averaged C stocks will ultimately define, on the long run, the entire OP ecosystem (biomass+soil) capacity to act either as a C sink or source.

### 2.4 Laboratory analysis

Soil particle size was determined by the pipette method after removal of the organic fraction with 30% H_2_O_2_ (Gee & Bauder, 1986). Soil pH was measured in water on a 1:2.5 (soil:water) suspension with a glass electrode. Soil available P was determined according to Bray II (Bray & Kurtz, 1945) and P in the extracts was determined colorimetrically. Available cations including Ca, K, Na and Mg were analyzed with Mehlich‐III solution (Mehlich, 1984) and horizontal shaking for 5 min followed by inductively coupled plasma spectrometry (PerkinElmer). Exchangeable acidity (EA) was determined extracting 2 g of dry soil with 10 ml of 1 M KCl, on a reciprocal shaker for 30 min. The extracts were then allowed to settle for 30 min and filtered three times with 30 ml of 1 N KCl. After adding phenolphthalein, final extracts were titrated with 0.01N NaOH. Effective cation exchange capacity (ECEC) was obtained by summing the amount of charge per unit soil of major cations (Ca, K, Na and Mg) and EA. Base saturation resulted from the division of the total sum of charge per unit soil of Ca, K, Na and Mg by ECEC. Total C and N contents, ^13^C and ^15^N were measured on oven‐dried at 35°C and ground soil encapsulated in tin cups with an Elemental Analyser (Eurovector) coupled to an isotope ratio mass spectrometer (Delta plus; Thermo Fisher) at the Center for Stable Isotope Research and Analysis (KOSI) of the University of Göttingen, Germany. Samples were free of carbonates and therefore total C content is equivalent to SOC. Soil biological analyses were conducted on topsoil (0–5 cm) and subsoil (50–70 cm) samples of three management zones (W, IZ and F). Samples of the H zone were not considered due to the similar chemical conditions that they shared with IZ samples. Measured microbial parameters included microbial biomass C (MBC), microbial biomass N (MBN), basal respiration, potential activity of four extracellular enzymes. Analyses were conducted following a 7‐day incubation period in the dark; therefore, our measured microbially mediated functions could differed from direct field measurements. Twenty grams of sieved and 35°C oven‐dried soils were rewetted to reach 60% water‐holding capacity and incubated at 24°C in 250 ml glass bottles closed with rubber septa. Also, dissolved organic C (DOC), ammonium (${\text{NH}}_{4}^{+}$) and nitrate (${\text{NO}}_{3}^{‐}$) were measured following this incubation.

Basal respiration measurements were performed by injecting air samples taken from the headspace air in the glass bottles with an airtight syringe into an infrared gas analyzer (EGM‐4 PP Systems). For this, the instrument's static sampling mode was set. Following headspace samples collection, CO_2_‐free air was flushed into the glass bottles. Soil MBC and MBN were determined on the incubated soil with the chloroform fumigation‐extraction method using an extraction coefficient of 0.45 for calculating MBC (Beck et al., 1997) and of 0.54 for MBN (Brookes et al., 1985). The DOC derived from soluble extracted C with K_2_SO_4_ on the non‐fumigated samples. For extraction of inorganic N (${\text{NH}}_{4}^{+}$ and ${\text{NO}}_{3}^{‐}$), 5 g of incubated soil was extracted with 30 ml of 1 M KCl and measured with an automated analyzer (SEAL AA3 HR Autoanalyser) and results expressed as mg kg^−1^ oven‐dried soil. To estimate soil microbial functioning, two indices were calculated. The microbial quotient was estimated as the ratio of MBC to SOC and the metabolic quotient as the ratio of basal respiration to MBC. Potential enzyme activities involved in the degradation of C, N and P including those involved in degrading chitin (β‐1,4‐N‐acetylglucosaminidase), leucine amino acid (leucine aminopeptidase) and phosphate groups (acid phosphatase) were assayed by fluorometric microplate assay using a modified method (Marx et al., 2005). Briefly, 1 g of incubated soil was mixed and homogenized for 1 min with a vortex in 50 ml of milli‐Q water and then sonicated at 50 J s^−1^ for 2 min (Ultrasonic Disintegrator). All assays were performed in buffered conditions with 2‐N‐morpholino‐ethanesulfonic acid (MES) and Trizma for 4‐methylumbelliferone (MUB)‐ and 7‐amino‐4‐methycoumarin (AMC)‐based substrates, respectively. For each sample, three aliquots of 50 μl of the stirred suspension were mixed in a 96‐well microplate with either MES or Trizma buffer and 100 μl of substrate solution of increasing concentration. After 0.5 and 2 h of incubation in the dark, the fluorescence was measured on a fluorometric microplate reader (BioTek SynergyMX). To quantify product release and account for quenching effects, a set of standards was prepared using MUB and AMC mixed with soil extract. Enzyme activities were expressed as nmol of substrate (MUF and AMC) converted h^−1^ and g^−1^ (dry weight) of soil.

### 2.5 Soil C stocks

The calculated SOC stocks resulted from the product of soil bulk density, layer thickness and SOC concentration. For SOC stocks calculations in the entire soil profile in each management zone (0–70 cm), a soil mass correction step was introduced to reduce potential confounding effects due to land‐use change or management zones. For this, the site with the lowest total soil mass in the IZ was considered, OP‐27 years, and all other cores were adjusted to this reference soil mass (Poeplau & Don, 2013). Since SOC stocks were calculated for each management zone, calculations were performed using the corresponding C content, bulk density and layer thickness of each management zone in each OP site. For upscaling to the plot level, the proportion that each management zone occupies at the plot level was considered (Table 1). Therefore, a weighted average mean considering the surface occupied by the four management zones was used (W = 12%; IZ 38%; F = 11% and H = 39%).

To quantify the contribution of OP‐derived C and savanna‐derived C to SOC stocks, changes in soil δ^13^C signatures were used in a two sources linear isotopic mixing model (Balesdent et al., 1987):

$$f=\frac{\left(\delta_{\text{sam}}‐\delta_{\text{ref}}\right)}{\left(\delta_{\text{op}}‐\delta_{\text{ref}}\right)},$$

where *f* is the relative proportion of OP‐derived C in SOC stocks. δ_sam_ is the δ^13^C of the soil sample at a given layer, δ_ref_ is the δ^13^C of the corresponding soil depth from savanna reference soil (C_4_) and δop is the δ^13^C of nine fine root biomass C samples (Rüegg et al., 2019). Relative proportions of OP‐derived C and savanna‐derived C were also calculated for each management zone.

### 2.6 Data analysis

The study was laid out in a type of split‐plot design, with Age as main plot factor and Management type (e.g., management zones) as subplot factor. There were six main plots repeated over 5 ages (0, 8, 12, 23 and 27 years). Each main plot is subdivided into four subplots, each with one of the four levels of the factor Management. All statistical tests were carried out with RStudio statistical software version 3.4.0 (R Core Team, 2017). General linear models were used to quantify the effects of management and time after savanna conversion on all measured biogeochemical variables. Simple linear regressions were performed to test the individual effect of time after savanna conversion (disregarding management zone) on bulk SOC stocks, OP‐derived and savanna‐derived SOC. For this, the linear model “lm” function was used.

Analysis of covariance tested the individual and combined effects of time after savanna conversion and management (quantitative and categorical explanatory variables, respectively) on all measured biogeochemical variables and SOC stocks using nested linear mixed models. These linear models allowed to represent the study design condition of having four management zones nested within each of the sampled OP plots. Such nested structure in the sampling design was included in the random effects part of the model with management (categorical explanatory variable with four levels: W, F, IZ and H management zones) nested within study sites (categorical explanatory variable with six levels: four OP plots and two savannas). Management and time after savanna conversion (quantitative variable) were fixed factors. The linear mixed effect “lme” function of the nlme package was employed to fit the models. Models’ residuals were assessed to ensure normal distribution and homogeneity of variance. If linear model assumptions were violated, then permutation analyses (Monte Carlo resampling tests) were conducted for the selected linear mixed‐effects model. When the within models were used, the “lstrends” functions in the LSMEANS package were used to test for the significance of the slopes of each management zone, where significance was declared using 95% confidence intervals (CI 95%) when the intervals did not overlap with zero. Slopes of management zones were further assessed using lsmeans pairwise comparison *t*‐test derived from lme. For all statistical tests, significance was declared at *p* < .05 and were evaluated using ANOVA type II sum of squares (car package). If not specified, all discussed differences are significant at *p* < .05.

A multivariate approach was used with a redundancy analysis (RDA) and Monte Carlo permutation test (999 permutations) to determine relationships between soil biogeochemical properties and explanatory variables (time after savanna conversion and management zones). RDA was performed using the “rda” function. Canonical variation partitioning with the “varpart” function was performed to assess the individual and collective contributions of time after savanna conversion and management to the total variation in soil biogeochemical variables.

## 3 RESULTS

### 3.1 Ecosystem C stocks

**FIGURE 1:**
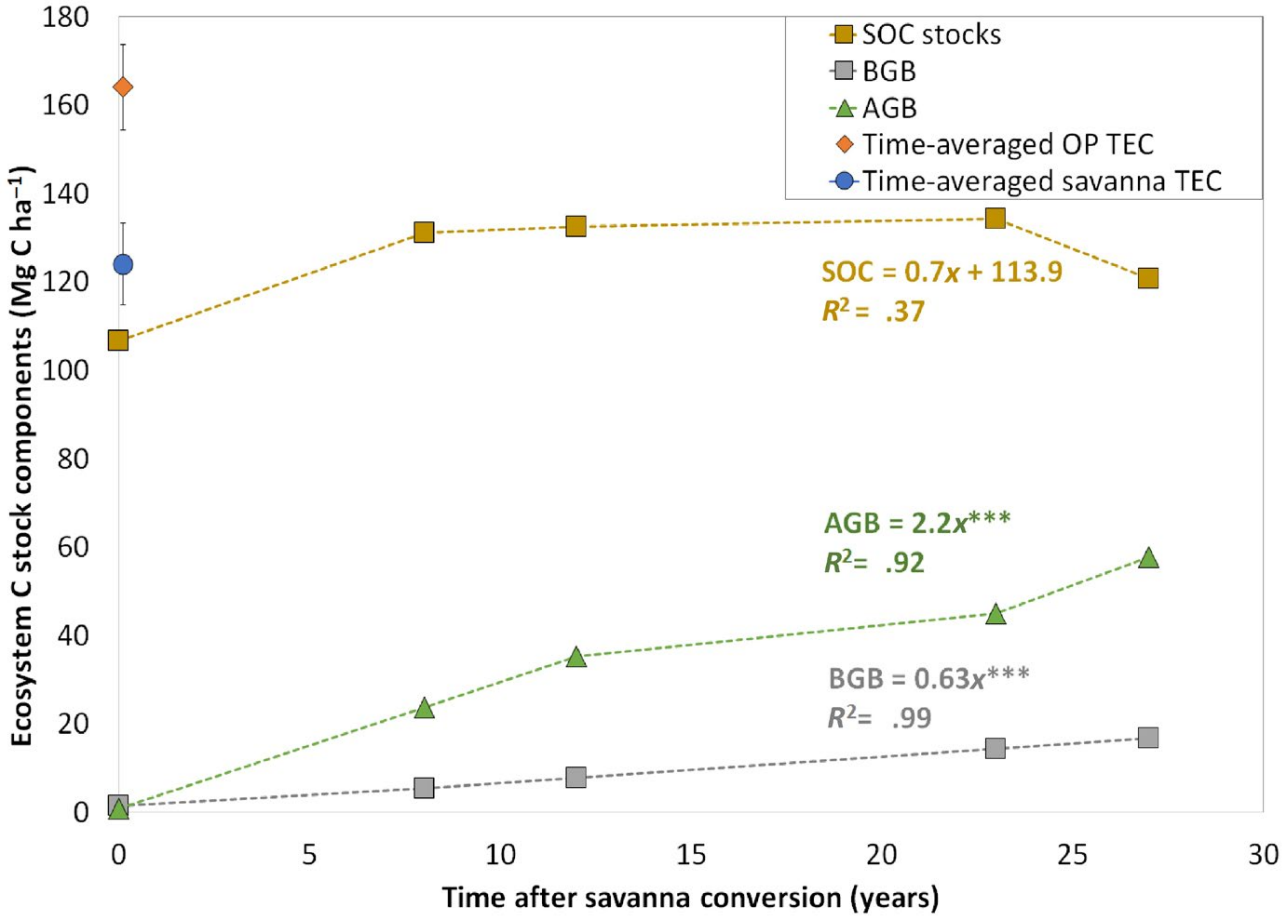
Ecosystem C stocks components in oil palm (OP) plantations and time‐averaged total ecosystem C stocks in OP plantations (orange diamond) and in the savanna (blue circle) over an OP‐rotation cycle, including soil C stocks down to 70 cm. Vertical bars around each total ecosystem C stock point represent SE. Soil organic C accumulation rates (Mg C ha^−1^ year^−1^) in the whole soil profile, aboveground biomass (ABG) and belowground biomass (BGB) are indicated by the equations that correspond to linear regressions for the entire plantation cycle

The studied savanna conversion into OP plantations increased total ecosystem C stocks aboveground and belowground biomass, and SOC. Even though OP plantations develop large biomass, the soil was the major C pool holding between 82% in the 8‐year‐old and 62% in the 27‐year‐old plantations of the total C in OP agroecosystems (Figure 1). Total OP biomass (aboveground+belowground biomass) increased linearly with cultivation time at a rate of 2.8 ± 0.17 Mg C ha^−1^ year^−1^, corresponding to a time‐averaged OP biomass C stocks of 42.2 ± 2.51 Mg C ha^−1^. Such time‐averaged OP biomass C stocks were about 20 times higher compared to savanna total biomass C stock, 2.1 ± 0.11 Mg C ha^−1^. Thereby, the increase in OP total ecosystem C stocks compared to savanna can be attributed mainly to the striking increase in total OP biomass with time.

Time‐averaged total ecosystem C stocks down to 70 cm depth were of 164.1 ± 9.6 and 124.0 ± 9.3Mg C ha^−1^ in OP plantations and in the savanna, respectively, thus savanna conversion into OP plantations implied a positive ecosystem C balance with a net gain of 40.1 ± 13.4 Mg C ha^−1^ (Figure 1). The estimated additional C storage potential was 147.2 Mg CO_2_ eq. ha^−1^.

### 3.2 Soil C stocks dynamics

**FIGURE 2:**
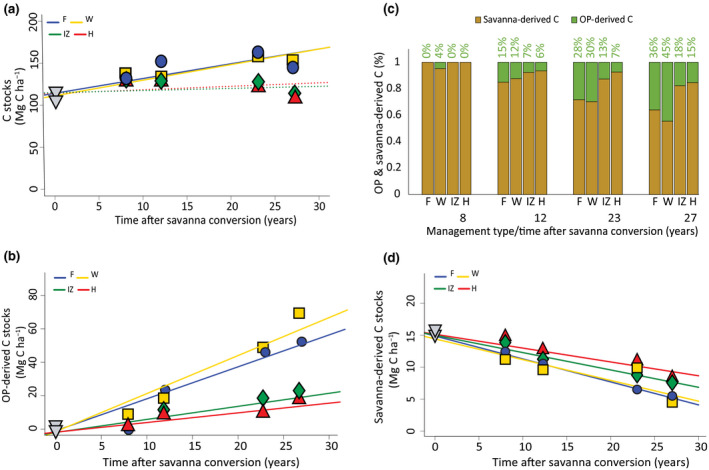
Soil C dynamics in oil palm plantations established on former savanna areas from. (a) Soil C stocks for each management zone over time in the full profile (0–70 cm); (b) Oil palm‐derived C accumulation according to management zone over time in the full profile; (c) Proportion of oil palm‐derived (numbers on top of the bars) and savanna‐derived C for each management zone over time in the full profile and (d) Savanna‐derived C dynamics at each management zone in the topsoil (0–5 cm). Management zones are: F, frond pile; W, weeded circle; IZ, interrow and H, harvest path. Solid lines represent significant changes with time

Bringing a former savanna into OP cultivation did not alter SOC stocks at the plantation scale after 27 years in the soil profile down to 70 cm. Mean SOC stocks in savannas and OP sites were of 106.6 ± 4.0 and 129.6 ± 3.0 Mg C ha^−1^, respectively (Figure 1). However, stock increases of 1.9 ± 0.3 Mg ha^−1^ yr^−1^ and 1.8 ± 0.6 Mg ha^−1^ yr^−1^ were found in management zones with high fertilizers and root inputs (W) and large crop's residues inputs from pruned OP fronds (F), respectively (Figure 2a). By contrast, SOC stocks remained constant at areas with low (IZ) and no inputs (H; Figure 2a; Table 1). Because the small share that the W and F areas represent within a plantation (23% together), the overall SOC stocks did not increase (Figure 1). In W and F, this SOC dynamic over time was very similar in each of the three uppermost surface soil layers (0–5, 5–10 and 10–20 cm; Table S1). At the three deepest soil layers (20–30, 30–50 and 50–70 cm depths), changes in SOC stocks due to savanna conversion or management zones were not detected (Table S1).

Management zones also affected the dynamics of both OP‐ and savanna‐derived C with cultivation time. In the entire soil profile, the accumulation of OP‐derived C under high fertilizers (W) and large crop residues (F) inputs followed the same pattern as the bulk OP‐derived SOC stocks at these same areas (Figure 2b). In spite of the absence of detectable significant change in bulk SOC at the low‐(IZ) and no‐input (H) areas, OP‐derived C increased slightly in these MZs at similar rates, substituting savanna‐derived SOC. New OP‐derived C accumulation rates were of 2.4 ± 0.3, 2.1 ± 0.3, 0.8 ± 0.2 and 0.6 ± 0.2 Mg ha^−1^ year^−1^ in W, F, IZ and H, respectively (Table 1). Nevertheless, total SOC consisted predominantly of savanna‐derived C across all the study time. The proportion of OP‐derived C in the total SOC pool across management zones was very modest, but increased with age of the plantation (Figure 2c).

At the large crop residue (F) and high fertilizers (W) input areas, the proportion of new OP‐derived C in the full profile was as high as 36% and 45%, respectively, after 27 years of OP cultivation (Figure 2c). The uppermost soil layer (0–5 cm) changed from predominantly savanna‐derived C to OP‐derived C after 27 years of OP cultivation at high‐input areas (W and F), but at low‐ and no‐input areas nearly 50% of the total C was still of savanna origin (Table S2). At the four subsequent soil layers (5–10; 10–20; 20–30 and 30–50 cm), OP‐derived SOC stocks increased under F and W with time, but no or minor changes were detected at the IZ and H areas. Interestingly, in the deepest soil layer (50–70 cm), new OP‐derived C increased with time at all four management zones (Table S3), but this had no effect on the bulk SOC stocks. At this same layer, the OP‐derived C accumulation rate in the high fertilizer input area was the highest, 0.13 ± 0.01 Mg ha^−1^ year^−1^, followed by the large crop residue input area, 0.08 ± 0.02 Mg ha^−1^ year^−1^. On the other hand, savanna‐derived C stocks decreased in the three uppermost soil layers but decomposition rates were not significantly affected by management zones (Figure 2d; Table S3).

### 3.3 Soil chemical and biological properties

**FIGURE 3:**
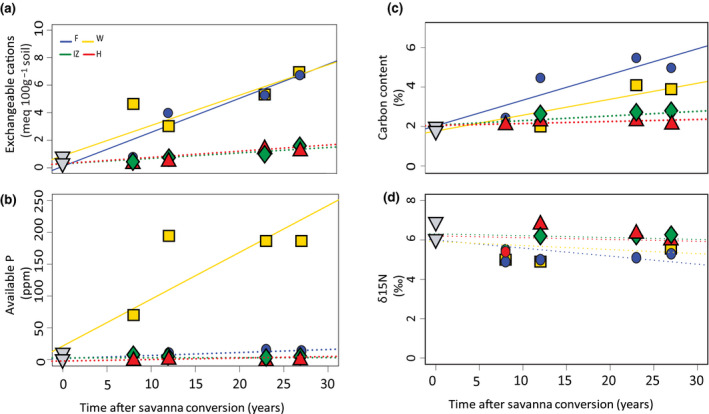
Soil nutrient dynamics in oil palm plantations established on former savanna areas in the 0–5 cm layer. (a) Sum of cations; (b) Available Bray‐P; (c) soil C content; (d) N signature. Management zones are: F, frond pile; W, weeded circle; IZ, interrow and H, harvest path. Solid lines represent significant changes with time

Overall, the regular application of fertilizers and crop residues led to a positive effect on soil surface (0–5, 5–10, 10–20 and 20–30 cm layers) nutrient dynamics (Table S4). Sum of cations (Ca, Mg, K, Na) values for rates of change were the greatest in areas receiving high fertilizer and large crop residues inputs at all soil layers. In the top 5 cm soil layer, these two input‐receiving areas contained about 20 times more base cations than the native savanna after 27 years of OP cultivation (Figure 3a). At the other two areas with low‐ and no‐input applications, changes in exchangeable cations in the top 5 cm were not detected over time. However, at all four layers below 10 cm, except at the 30–50 cm layer, the sum of cations values increased only due to time after savanna conversion and not in relation to management zones (Table S4).

Not only major cations, but also available P increased with OP cultivation time at areas receiving high fertilizer inputs (Figure 3b). However, this increase in available P was not observed at F. This available P improvement occurred down to 30 cm depth but not deeper (Table S4). Similar to the dynamics of exchangeable cations, the low‐ and no‐input areas did not vary in available P content between management zones at any soil layer. Soil C content increased in a similar fashion at the three uppermost soil layers over time at areas with high fertilizer and large crop residues inputs. At areas with low and no inputs, changes over time were not detected. Also, differences in C content between management zones increased with time after savanna conversion. Topsoil C content ranged from 2.1% to 5.5% across all management zones and in the natural savannas. At areas with large crop residues and high fertilizer inputs, soil C content peaked at 23 years after savanna conversion by about threefold and twofold, respectively (Figure 3c).

There was no effect of time and management on soil pH down to 30 cm depth. However, in the area subjected to high fertilizer input, there was an acidification at the 30–50 cm soil layer (Table S4). Management zones altered isotopic N dynamics. Unexpectedly, soil δ^15^N signatures decreased progressively with OP cultivation time at areas with large crop residues inputs down to 10 cm depth (Figure 3d). Although not significant, at areas subjected to high fertilizer input there was also a declining trend as in areas with large crop residues inputs (Table S4).

In general, the majority of soil microbial traits were influenced either by management, time after savanna conversion or both in the topsoil (0–5 cm; Table 2) and few or no effects were detected in the subsoil (data not shown). The soil surface of areas, where crop residues were applied (F), enable the highest biological activity. There, microbial respiration, ${\text{NH}}_{4}^{+}$ production and enzymatic activities of leucine had the highest values. Microbial respiration also increased with OP cultivation time at the surface of soils with high fertilizer inputs (Table 2). While soil MBC decreased at areas with low‐ (IZ) and high fertilizer inputs (W), no changes were detected with time at areas with high crop residue inputs (F; Table 2). Irrespective of management zones, the microbial and metabolic quotients decreased and increased, respectively, with time after savanna conversion.

**TABLE 2:** Temporal rate of changes and SE in soil biological variables in the topsoil (0–5 cm) over one OP rotation cycle. Results of ANCOVA linear mixed models. *p*‐values are indicated with the respective symbols *** for *p* < .001, ** for *p* < .01, * for *p* < .05 and NS for *p* > .05

Soil properties	Unit	Time effect	Management effect	Management/time	Management practice		
					W	IZ	F
Microbial respiration	mg C kg^−1^ soil day^−1^	NA	NS	***	0.84 (0.14)**	NS	1.30 (0.29)*
Microbial biomass C	mg kg^−1^	NA	NS	***	−6.79 (2.3)*	−8.07 (1.59)**	NS
Ammonium	mg kg^−1^	NA	NS	***	NS	NS	5.02 (0.93)**
Leucine aminopeptidase	nM MUC g^−1^ h^−1^	NA	NS	***	NS	−0.98 (0.54)*	2.11 (0.78)*
Microbial quotient	NA	−0.41 (0.05)***	NS	NA	NA	NA	NA
Metabolic quotient	NA	0.009 (0.002)***	NS	NA	NA	NA	NA
DOC	μg C g^−1^	NS	*	NA	NA	NA	NA
Phosphatase	nM MUF g^−1^ h^−1^	NS	*	NA	NA	NA	NA

Note

Values are rates of change over one OP cultivation cycle (27 years) with standard errors in parentheses. When the interaction between management and time after savanna conversion (fixed effects) was not significant, the additive model (management+time after savanna conversion) was used, but if the interaction between the fixed effects was significant, then the within model that tested for the effect of time after savanna conversion within management was used.

Abbreviations: DOC, dissolved organic carbon (K_2_SO_4_‐extract);NA, not applicable; OP, oil palm.

**FIGURE 4:**
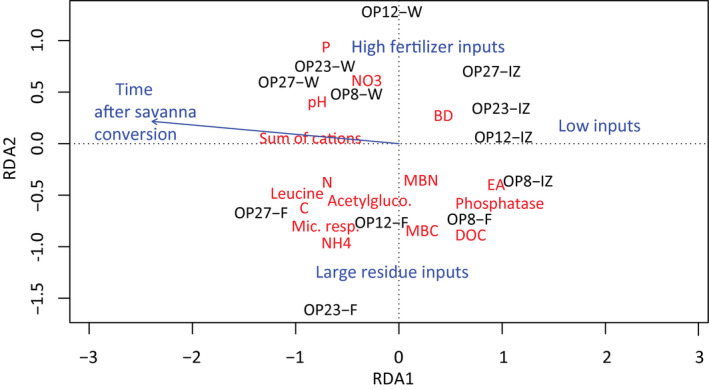
Redundancy analysis (RDA) of soil biogeochemical variables in the topsoil (0–5 cm) constrained for time after savanna conversion and management zones. The OP plots are indicated as OP##‐X, where ## is the years after savanna conversion and X is the management zone; large residue inputs (F), high fertilizer inputs (W) and low inputs (H). BD, bulk density; DOC, dissolve organic C; EA, exchangeable acidity; ${\text{NH}}_{4}^{+}$, ammonium; ${\text{NO}}_{3}^{‐}$, nitrate; MBN, microbial biomass N; MBC, microbial biomass C; Mic. resp, microbial respiration; P, available phosphorus (Bray P)

The relative role of time after savanna conversion and management zones (W, F and IZ) on soil biogeochemical properties were quantified and synthesized in a multivariate analysis. RDA showed that regardless of time after savanna conversion into OP, soils of each management zone cluster together, forming three well‐defined groups characterized by specific biogeochemical properties (Figure 4). Both axes 1 and 2 were statistically significant and accounted for 32.8% and 22.7% of the total variation in soil biogeochemical properties, respectively. Overall, areas with high fertilizers (W) and large crop residue (F) inputs favored either high soil fertility or high soil biological activity. More specifically, the area with large crop residue inputs had the highest soil biological activity, that is, MBC, microbial respiration and potential enzymes activity, and the area with high fertilizer inputs had the highest soil fertility, that is, sum of cations, P and ${\text{NO}}_{3}^{‐}$ (Figure 4). Management explained much more variation (44%) in soil biogeochemical properties than time after savanna conversion (9%).

## 4 DISCUSSION

The prevailing notion that converting natural ecosystems into cultivated land inevitably and negatively affects ecosystem C stocks does not always hold, as demonstrated in this study of savanna conversion into OP. Our study is the first to present evidence that converting savanna to OP can increase ecosystem C storage across a full cultivation cycle. According to the first hypothesis, savanna conversion into OP resulted in a positive ecosystem C balance (40.1 Mg C ha^−1^), with potential to be further enhanced if better soil management practices are adopted. This contrasts with other land‐use change trajectories, in particular with the deforestation scenario where it has been shown that expansion of OP plantations on forested areas led to striking ecosystem C losses, amounting to 173 Mg C ha^−1^ (Guillaume et al., 2018). While deforestation usually results in large total ecosystem C losses (total biomass and SOC), other deforestation‐free alternatives, such as pasture conversion to OP, result in trade‐offs between major total ecosystem C components, where gains in aboveground biomass are offset by losses of pasture‐derived soil C, resulting in overall neutrality in terms of ecosystem C balance (Quezada et al., 2019). SOC loss did not occur in this study because there was very little decomposition of savanna‐derived C, that was compensated by OP‐derived C at all soil layers, resulting in a substantial net positive ecosystem C outcome for this land‐use change trajectory because of the C storage gain in OP biomass (Table S3). The values found for aboveground and belowground biomass stocks, rates of change and time‐averaged stocks in OP and savanna are similar to previously published results (Guillaume et al., 2018; Kotowska et al., 2015; Ramírez‐Restrepo et al., 2019; Ziegler et al., 2012).

Other studies of grassland conversion to OP plantations have shown broadly similar results with little SOC losses. Increased bulk SOC stocks and no losses of grassland derived C were reported over a 25‐year OP chronosequence established on former grasslands in Papua New Guinea (Goodrick et al., 2015), partially explained by black C produced during grassfires. In our study, the relatively slow savanna‐C decomposition over time could be explained by physical stabilization of old savanna C due to high content of minerals that protected SOC from mineralization, that is, clay, iron and aluminum oxides and hydroxides, as suggested in other studies in tropical areas (Baldock & Skjemstad, 2000; Barthès et al., 2008). Indeed, it has been shown that poorly crystalline Fe‐ and Al‐oxides play an important role in stabilizing SOC in OP plantations derived from grasslands (Goodrick & Nelson, 2017). In the same region, the more productive grasslands with higher initial SOC that developed on soils with lower clay content experienced SOC losses during land‐use change in OP (Quezada et al., 2019). Similarly, in another study also conducted in the region of Los Llanos, reduced SOC stocks in a 9‐year‐old plantation with sandy soils were found (Rüegg et al., 2019). In Brazil, a study reported lower SOC stocks in OP plantations of pasture origin after 8 years of cultivation, but no information is given with respect to soil mineral content (Frazão et al., 2013).

Our results point to the potential for adopting soil management practices that can increase soil nutrients availability and organic matter, which, in turn, can enhance the potential of OP plantations in savanna regions to act as C sinks. This could be possible if the benefits of increased SOC stocks, enhanced fertility and soil microbial activity observed under the W and F management zones, could be extended in the plantation. In partial support to our second hypothesis, not only the application of crop residues in F, but also chemical fertilizers in W, where root density is highest, led to SOC accumulation rates in the whole soil profile, between 1.8 and 1.9 Mg C ha^−1^ year^−1^. Other studies have found highly variable C and N stocks between management zones, with the F area being the one with the highest SOC stocks (Frazão et al., 2013, 2014; Khasanah et al., 2015; Law et al., 2009). Such accumulation rates were similar to that found for increased aboveground biomass in OP plantations in one full rotation cycle, 2.2 Mg C ha^−1^ year^−1^ (Figure 1).

The accumulation of bulk SOC stocks was prompted by substantial increases in OP‐derived C that largely surpassed the decomposition of savanna‐derived C in those zones (Table S3). As found in other studies (Carron et al., 2015; Frazão et al., 2013; Rüegg et al., 2019), areas receiving high fertilizer inputs (W) had the highest SOC stocks. The SOC increase we observed at W is related to fine roots that have their highest density near the tree and which could have been stimulated by the high nutrient supply, so greater belowground organic matter inputs (Goodrick et al., 2016; Rüegg et al., 2019). This is of relevance, because C stored in soils is less vulnerable to losses compared to biomass C storage. Therefore, management of external inputs plays an important role in enhancing the C storage capacity of OP agroecosystems so that the C benefits of savanna conversion could result not only in increased biomass C, but also in a simultaneous gain in SOC stocks. According to the rates of C accrual in fertilizers and crop residues input zones, this could further increase C storage potential of savanna conversion.

Increases in bulk SOC stocks due to organic C or fertilizer inputs were limited to the three uppermost soil layers. This compares to a recent global meta‐analysis, where the effects of cover crops were limited to the top 30 cm (Poeplau & Don, 2015), which could be explained by reduced C and nutrient inputs into subsoil layers. We did not see any sign of SOC stocks approaching a new steady state in the top 20 cm when inputs where applied, in contrast to other studies where SOC storage capacity becomes smaller with time after land‐use change or the implementation of better management practices such as reduce tillage (Sommer & Bossio, 2014; West & Six, 2007).

In contrast to the similar rates of C accumulation observed at input receiving areas W and F, SOC stocks remained unchanged with cultivation time in areas away from palms where belowground inputs are reduced and aboveground inputs absent (IZ and H), despite IZ receiving nutrients during the fertilization process (Table S1). All these factors together could indicate that both organic matter and nutrients inputs benefited synergistically to soil C accumulation at the W and F zones. This is in agreement with the findings in a mature plantation in the same savanna area where it was demonstrated that the positive effects of nutrients on SOC accumulation were driven by C inputs via OP roots (Rüegg et al., 2019). Moreover, input additions in our study resulted in accumulation rates greater than those reported in long‐term and meta‐analysis studies where conservation agriculture practices such as reduced tillage, residue retention and use of cover crops were implemented (Batlle‐Bayer et al., 2010; Lal et al., 2015; Mazzoncini et al., 2011; Tautges et al., 2019).

The hypothesis that microbial activity would be enhanced along with soil fertility was only partly confirmed. We found that SOC pools, such as microbial C, decreased at W and remained unchanged at F, although potentially mineralizable C, measured by soil respiration, and enzyme activity were higher at F than at W and in savannas. This is in line with other studies where high‐resolution field measurements of soil CO_2_ emissions revealed that soil CO_2_ emissions were the highest at F, intermediate at W and the lowest at IZ (Goodrick et al., 2016). In general, adding crop residues at F supported a level of biological activity similar to that in natural savannas. This is an important perspective, because managed agroecosystems rarely maintain similar levels of soil biological activity compared to natural counterparts. Although we do not have direct evidence on the distribution of C between SOC fractions, we believe that the distribution of total SOC might vary among the various C pools. Further analyses of the soils used in this study with more advanced techniques, that is, SOC fractionation and isotopic tracer, would allow to have a better mechanistic understanding of SOC stabilization.

The application of large amounts of fertilizers in W resulted in reduced soil microbial activity, but increased soil nutrient availability (i.e., P, ${\text{NO}}_{3}^{‐}$) compared to the application of crop residues at F that induced soil conditions favoring soil microbial activity (i.e., soil respiration, enzymatic activities). It is possible that the increase with time after savanna conversion of the metabolic quotient, at all management zones, indicates of a shift in soil microbial community structure, for example, bacterial:fungal ratios. Deeper investigations on soil microbial communities at the scale of management zones would be very useful. To date, such studies in OP have used composite samples from all management zones, therefore virtually no data exist on soil microbial populations at each management zone. The significant variations in most soil properties among management zones and with time after savanna conversion, contrast with results from another study in grassland conversion where almost no changes in soil properties were reported after 25 years (Nelson et al., 2014). This highlights trade‐offs between agronomical management purely based on fertilizers addition and soil biological functioning. Furthermore, it has been demonstrated that the accumulation of OP fronds on the soil surface is not an efficient practice for SOC stabilization and that the positive effects on SOC stocks result mainly from the improved soil conditions, such as nutrient availability and moisture, under the decomposing fronds (Rüegg et al., 2019). We draw similar conclusions because if accumulation of C from OP fronds (F) would be efficient, then soil C accumulation rates would be higher at F than at the area close to the trunk (W), which was not the case.

A striking outcome of our work is that management (as reflected by management zones) had about five times more explanatory power with regard to soil biogeochemical variations as did land‐use change from savanna to OP. The magnitude and direction of changes in soil biogeochemical properties were more related to specific management interventions within plantations than land‐use change conversion per se. This suggests opportunities for improving OP cultivation sustainability through management interventions. For example, recent on‐farm experiments have shown that understory vegetation in OP plantations can improve various environmental aspects without affecting productivity (Ashton‐Butt et al., 2018; Rochmyaningsih, 2019). We propose that instead of allowing a total cover of the OP plantations' understory surface area, the regrowth of natural vegetation should be focused on the IZ areas. This recovery of natural vegetation within plantations can serve as a buffer against biodiversity decline and thereby increase beneficial insects and mammal's species, with potential benefits for pest and disease control and thus reduction of pesticides applications. In addition, soil conditions could also be enhanced by increasing soil biodiversity and therefore soil nutrient cycling and SOC stocks. Application of compost from OP mill waste is already practiced in some farms, albeit it poses problems of profitability, in particular because of the transport. Alternatively, modifications in the timing, placement and integration of nutrients and organic matter sources, including plantation residues, should be considered. This could be achieved by the distribution of decomposing palm leaves to the area around the trunks (W), which would increase plant‐available nutrients and soil water content, boosting soil microbial activity in the OP rooting zone and therefore reducing the need of external inputs. The change of placement and integration of inputs should also be put in perspective with the change of understory managements in order to promote root development of natural vegetation in the IZ area. Given the inherent low nutrient content of savanna soils and tropical soils in general, the supply of organic matter should go hand in hand with a balanced supply of mineral nutrients via fertilizers so that substrate stoichiometry does not constrain soil microbial growth and activity (Kirkby et al., 2013). Partial replacement of synthetic fertilizers by organic nutrient sources such as compost might further reduce greenhouse gases, that is, N_2_O emissions, and increase soil aggregation (Mpeketula & Snapp, 2019; Tautges et al., 2019).

We provided a proof‐of‐concept that OP expansion in savanna regions can result in a positive ecosystem C budget. However, we recognize that our study has some limitations associated mainly with the small range of environmental conditions where the study sites were located. In our chronosequence approach, a common approach of numerous ecological studies, the site selection process sought to minimize the high heterogeneity among different savanna types to test the idea that alternative land‐use change pathways might have practical application from a carbon mitigation potential. We sampled in plantations sufficiently large with management and soil and climate conditions representatives of a complete OP cultivation cycle in the most dominant savanna type of the Colombian Llanos. Nevertheless, considering the limited availability of plantations with similar age in the study area, spatial replication had to be kept limited. In spite of these methodological limitations, the overall positive ecosystem C balance found in this study conforms with estimates of non‐empirical studies.

Our findings could foster complementary empirical studies that consider the heterogeneity of savanna ecosystems, particularly given edaphic and climatic variations so that regional estimates of ecosystem processes, that is, carbon sequestration and storage, can be generated. Beneficial ecosystem C outcome is highly relevant in the context of reducing C emissions from deforestation. It is, however, important to note caveats on the expansion of agriculture in savanna regions. Recent studies suggest both low and high impacts on savanna biodiversity due to its conversion into OP plantations in Los Llanos region of Colombia (López‐Ricaurte et al., 2017; Ocampo‐Peñuela et al., 2018; Prescott et al., 2016). While biodiversity losses associated with conversion of savannas might not be as high as losses resulting from forest conversion to OP, it nevertheless remains the case that natural savanna are highly threatened systems with a unique biodiversity. In this context, future expansion is not recommended on unique and valuable natural savanna ecosystems. Instead, OP expansion should take place on previously transformed lands in savanna regions such as degraded pastures which area abundant in the Neotropics (i.e., nearly 18 million ha only in Brazil; Pereira et al., 2018) or, when restoration is not viable, on degraded savannas. This would minimize the impacts on biodiversity and avoid the large ecosystem C losses and other environmental burdens of deforestation (Gilroy et al., 2015; Quezada et al., 2019). Finally, while we provide here some elements for a more sustainable OP agriculture, further research is warranted to quantify the complex interplay and outcomes that might exist between the recommended practices and the discussed deforestation‐free LUC alternatives on other important environmental aspects.

## CONFLICT OF INTEREST

The authors declare that the research was conducted in the absence of any commercial or financial relationships that could be construed as a potential conflict of interest.

## Supporting information

Supplementary Material

## Data Availability

Data supporting the findings of this study will be available on the Swiss Federal Research Institute (WSL) Digital Repository.
