# Supplementary material for: Deforestation‐free land‐use change and organic matter‐centered management improve the C footprint of oil palm expansion
Source: Glob Chang Biol. 2022 Jan 21;28(7):2476–90. doi: 10.1111/gcb.16069 (PMC9304317; doi:10.1111/gcb.16069)
Supplement: Supplementary file 1 — Supplementary Material [file GCB-28-2476-s001.docx]

**Supplementary information**


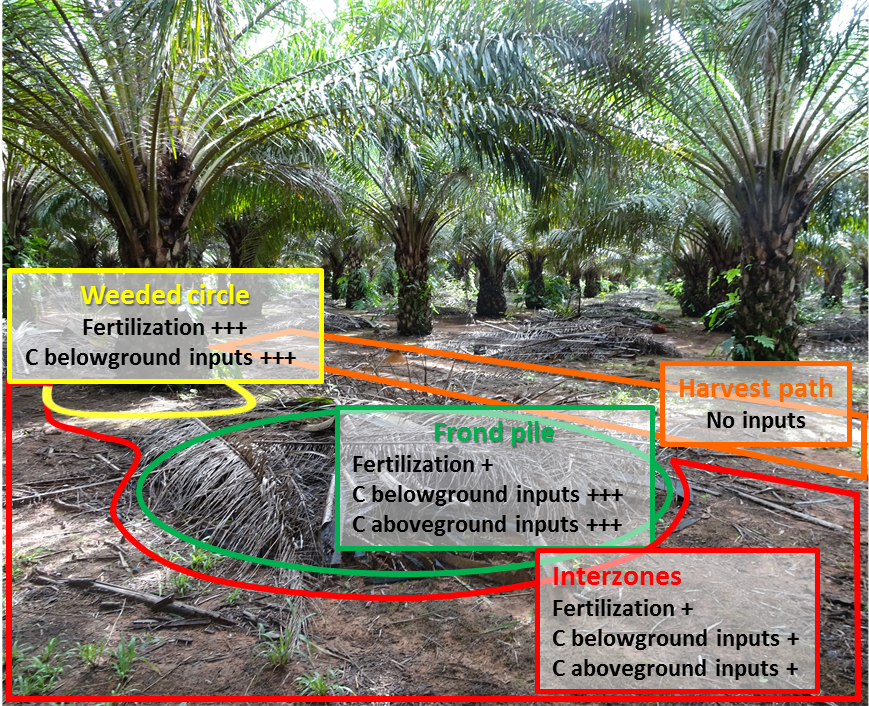


Supplementary figure 1. Schematic representation of oil palm management zones in a mature plantation (9 years old). + symbols refer to the magnitude of the input, *i.e.* fertilization +++, is high fertilizers application and fertilization +, is low fertilizers application.

Table S1. Rates of change in bulk SOC stocks (Mg ha^-1^ yr^-1^) and SE across one OP-rotation cycle (27-years) by layer. Results of ANCOVA using linear mixed-models. *P*-values are indicated with the respective symbols *** for *P*<0.001, ** for *P*<0.01, * for *P*<0.05, NS for *P*>0.05. NA stays for not applicable. When the interaction between time after conversion and management type was significant the model with the effect of time after savanna conversion within management type was used.


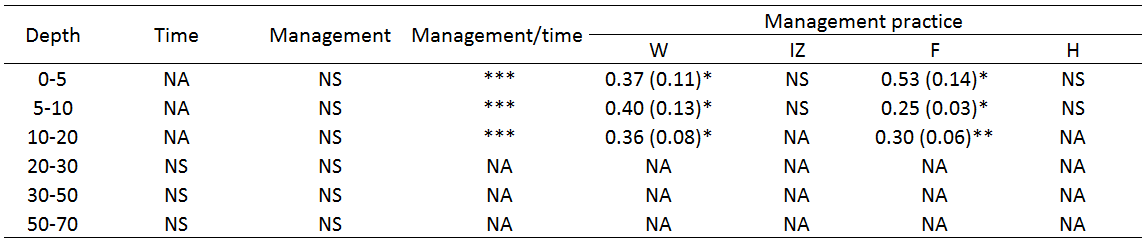


Table S2. Proportion (%) of OP and savanna-derived C by layer and time after savanna conversion at each management zone. See statistics of rate of changes in Table S3


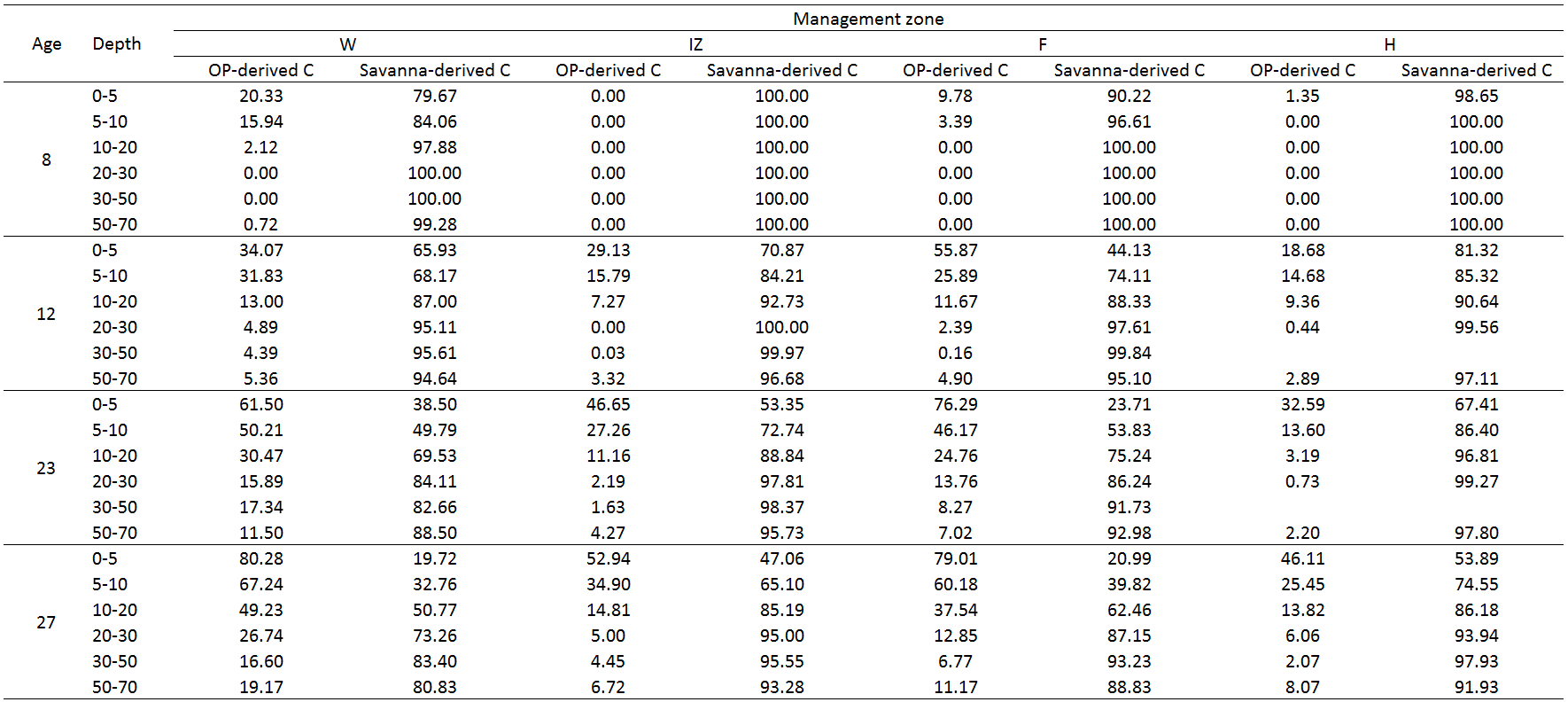


When values are 0 or 100, they were put as such due to slightly negative or beyond 100 values.

Table S3. Rates of change of OP- and savanna-derived C (Mg ha^-1^ yr^-1^) and SE across one OP-rotation cycle (27-years) by layer. Results of ANCOCA linear mixed-models. *P*-values are indicated with the respective symbols *** for *P*<0.001, ** for *P*<0.01, * for *P*<0.05, NS for *P*>0.05. NA stays for not applicable. When the interaction between time after conversion and management type was significant, the model with the effect of time after savanna conversion within management type was used.


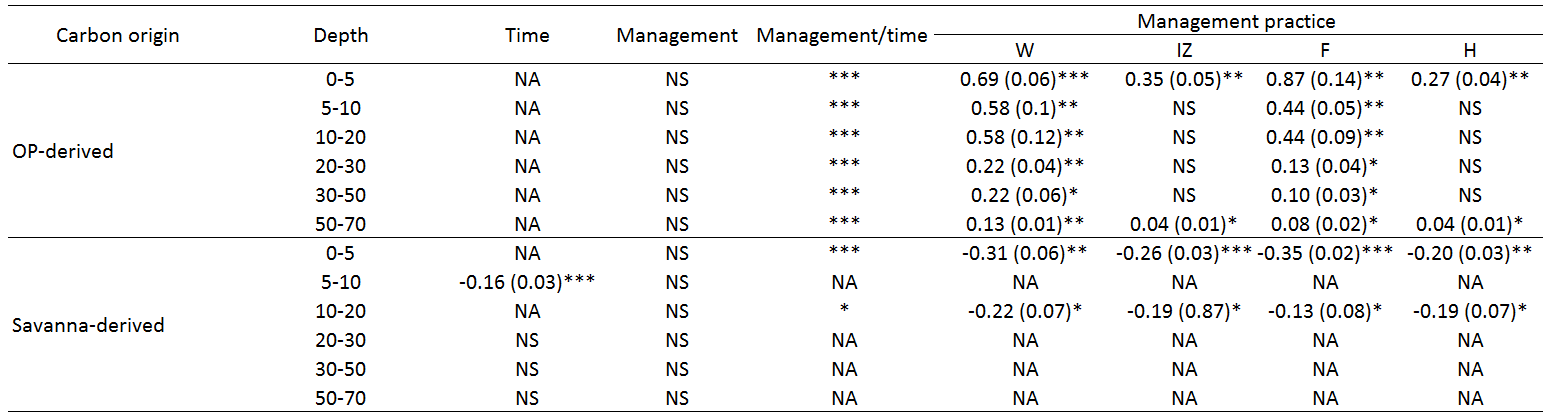


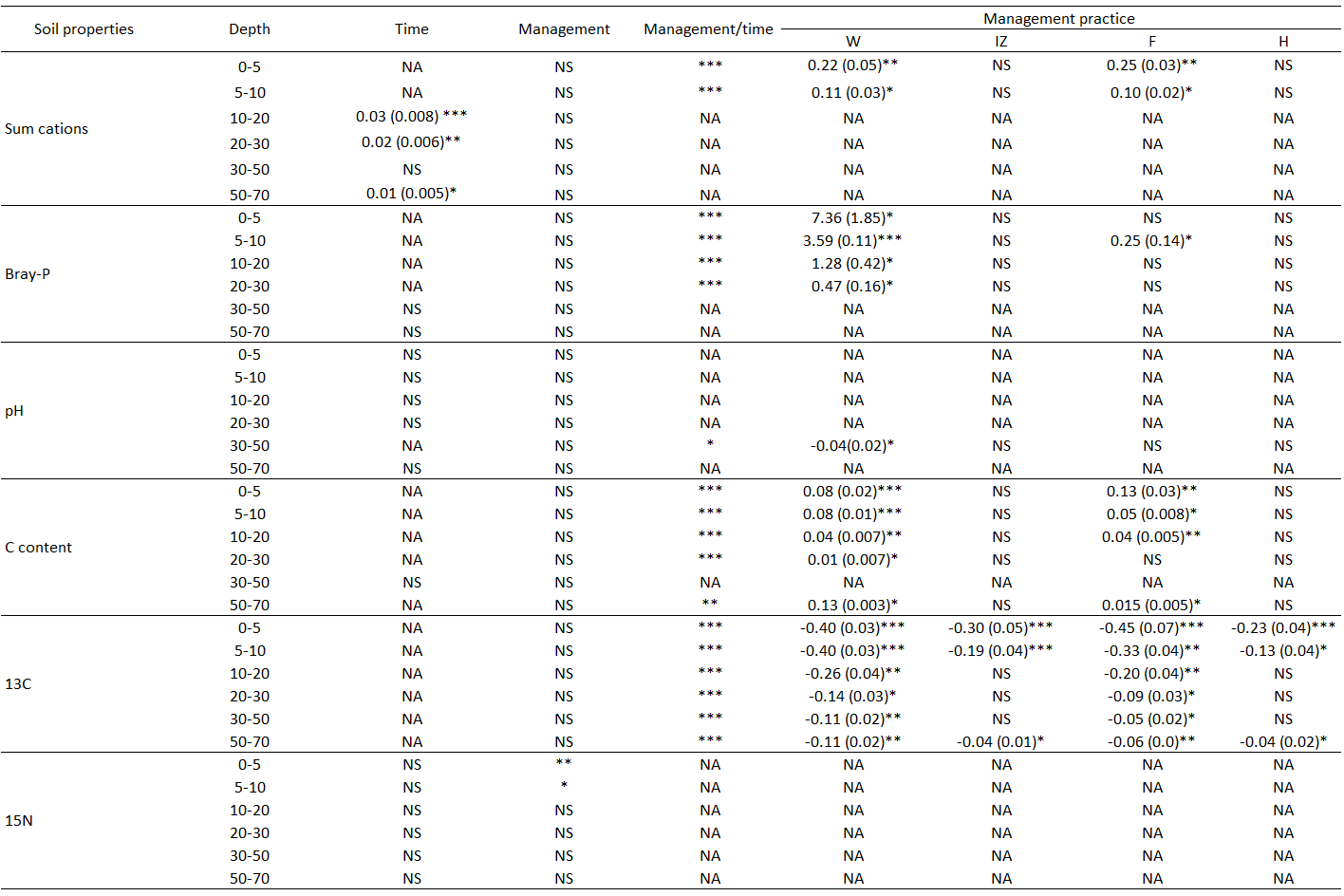
Table S4. Rates of change in soil chemical properties and SE within given soil layers over one OP rotation cycle (27-years). Results of ANCOCA linear mixed-models. *P*-values are indicated with the respective symbols *** for *P*<0.001, ** for *P*<0.01, * for *P*<0.05, NS for *P*>0.05. NA stays for not applicable. When the interaction between time after conversion and management type was significant, the model with the effect of time after savanna conversion within management type was used.
